# Advance in Iron Metabolism, Oxidative Stress and Cellular Dysfunction in Experimental and Human Kidney Diseases

**DOI:** 10.3390/antiox13060659

**Published:** 2024-05-27

**Authors:** Tiancheng Xie, Li Yao, Xiaogang Li

**Affiliations:** 1 Department of Internal Medicine, Mayo Clinic, Rochester, MN 55905, USA; xietiancheng192@gmail.com; 2 Department of Biochemistry and Molecular Biology, Mayo Clinic, Rochester, MN 55905, USA; 3 Department of Nephrology, The First Hospital of China Medical University, Shenyang 110001, China; 19922015@cmu.edu.cn

**Keywords:** iron homeostasis, oxidative stress, kidney disease, CKD, AKI, DKD

## Abstract

Kidney diseases pose a significant global health issue, frequently resulting in the gradual decline of renal function and eventually leading to end-stage renal failure. Abnormal iron metabolism and oxidative stress-mediated cellular dysfunction facilitates the advancement of kidney diseases. Iron homeostasis is strictly regulated in the body, and disturbance in this regulatory system results in abnormal iron accumulation or deficiency, both of which are associated with the pathogenesis of kidney diseases. Iron overload promotes the production of reactive oxygen species (ROS) through the Fenton reaction, resulting in oxidative damage to cellular molecules and impaired cellular function. Increased oxidative stress can also influence iron metabolism through upregulation of iron regulatory proteins and altering the expression and activity of key iron transport and storage proteins. This creates a harmful cycle in which abnormal iron metabolism and oxidative stress perpetuate each other, ultimately contributing to the advancement of kidney diseases. The crosstalk of iron metabolism and oxidative stress involves multiple signaling pathways, such as hypoxia-inducible factor (HIF) and nuclear factor erythroid 2-related factor 2 (Nrf2) pathways. This review delves into the functions and mechanisms of iron metabolism and oxidative stress, along with the intricate relationship between these two factors in the context of kidney diseases. Understanding the underlying mechanisms should help to identify potential therapeutic targets and develop novel and effective therapeutic strategies to combat the burden of kidney diseases.

## 1. Introduction

Kidney disease, another name for renal diseases, is a condition in which the kidney is injured and unable to function correctly. The kidneys function by filtering waste and excess fluids from the blood to form urine. When the kidneys are not functioning properly, waste products will accumulate in our body and cause various health problems [1]. There are different types of kidney diseases, including acute kidney injury (AKI) and chronic kidney disease (CKD) and diabetic kidney disease (DKD). AKI occurs suddenly and is often reversible with prompt treatment, while CKD develops over time and can lead to permanent kidney damage [2,3]. DKD is a kidney damage caused by diabetes, and is a specific type of CKD and one of the prevalent etiologies of CKD [4]. Diabetic patients develop DKD due to the effects of long-term high blood sugar and suffer from impaired kidney function. IgA nephropathy is also a type of CKD characterized by immune-mediated deposition of immune complexes in the mesangial cells of the kidneys, leading to chronic inflammation and kidney damage [5]. 

CKD and AKI are prevalent conditions with significant implications for public health worldwide. The intricate interplay of iron metabolism and oxidative stress is gaining significance in elucidating the pathophysiology and advancement of these renal diseases. Iron, a crucial micronutrient involved in numerous cellular functions, is tightly regulated in the body to maintain homeostasis [6]. A deviation from this fine equilibrium may result in abnormal iron levels, which can aggravate and accelerate CKD and AKI.

In CKD, the dysregulation of iron metabolism can have profound effects on renal function [7]. Iron overload, often observed in CKD patients undergoing hemodialysis, can promote oxidative stress via the overproduction of ROS [8]. ROS, in turn, can induce renal fibrosis and dysfunction, exacerbating the progression of CKD. Conversely, iron deficiency in CKD patients can impair erythropoiesis and exacerbate anemia, further complicating disease management [9].

Iron metabolism and oxidative stress also play a critical role in the pathophysiology of AKI. For example, ischemic AKI, a common form of AKI, is characterized by renal hypoxia and reperfusion injury, also resulting in the overproduction of ROS and oxidative stress [10]. The interaction of iron metabolism and oxidative stress in AKI can exacerbate renal injury and impair recovery, emphasizing the necessity of understanding the underlying mechanisms of acute kidney damage.

In this review, we discuss the roles and molecular mechanisms underlying iron dysregulation and oxidative stress in AKI, CKD and DKD. We also discuss the intricate relationship between iron metabolism and oxidative stress in kidney diseases. Understanding the molecular mechanisms underlying iron dysregulation and oxidative stress can aid in the identification of the prospective treatment targets and novel therapeutic techniques to mitigate renal damage and the process of kidney diseases.

## 2. The Regulation of Iron Metabolism

### 2.1. Iron Absorption from Guts to Plasma

After birth, human iron intake depends entirely on dietary iron, which consists mainly of heme iron and non-heme iron [11]. Of these, heme iron is mainly derived from hemoglobin and myoglobin contained in meat and fish. Non-heme iron comes mainly from vegetables and cereals [12]. Heme iron and non-heme iron are absorbed at different rates: heme iron has an absorption rate of approximately 20%, while non-heme iron typically has a lower absorption rate ranging from 2% to 5% [12]. Despite slight variations in the site of absorption, overall, dietary iron is absorbed in the small intestine [13]. Divalent metal transporter 1 (DMT1), located in the apical membrane of enterocytes, is capable of transporting non-heme iron from the intestinal lumen into the enterocyte via proton coupling [14]. However, DMT1 is only responsible for the transport of divalent metal ions; most of the iron in food is in the form of ferric ion. Thus, ferric iron must first be converted into ferrous iron at the brush border of the endocytes by a ferric reductase, duodenal cytochrome b (DCYTB) [15]. Iron is typically stored in ferritin within enterocytes [16]. Animal experiments indicate that in cases of iron deficiency, this iron is released into the plasma through ferroportin (FPN) with the assistance of hephaestin [17,18]. For heme iron, Majid Shayeghi et al. found that a membrane protein named heme carrier protein 1 (HCP1) is capable of its transportation into enterocytes [19]. 

### 2.2. Plasma Iron Transportation and Uptake by Other Cells

The transportation of iron throughout the body is facilitated by transferrin (TF), a protein that binds to iron tightly, allowing for uptake by all types of cells. This process requires recognition by transferrin receptor protein 1 (TFR1) [20], a type II dimeric transmembrane receptor [21]. TFR1 is located on all cell membranes and serves as the main receptor for iron entry into most cells. Human transferrin (hTF), a bilobal glycoprotein that is secreted from the liver into the bloodstream, has a high affinity for reversible binding of ferric irons [22]. It has been indicated that a deficiency in TF could lead to the development of anemia, iron overload in specific tissues, and potentially fatal outcomes [23]. TFR1 forms a 2:2 equimolar complex with hTF, with each TFR1 monomer binding to one molecule of hTF [24]. Then, the TF-TFR1 complex is internalized into cells through clathrin-mediated endocytosis [25]. Upon endosomal entry into the cell and subsequent removal of the clathrin proteins, protons flow inward into the endosome, which leads to the acidification of the endosomal environment. This, in turn, induces conformational changes in the complex to mediate the release of ferric iron [26]. The released ferric iron is subsequently reduced to ferrous iron by the six transmembrane epithelial antigen of the prostate family of reductases (STEAP) or by lysosomal cytochrome B (LCYTB), and transported to the cytoplasm by intracellular ferrous iron transporters including DMT1 and zinc transporter (Zip14) [27,28] in in vitro experiments. After releasing the iron, apo-TF/TFR1 complex (without TF-bound iron) returns to the cell surface and dissociates. 

TF-bound iron is generally considered as the main source of iron for most cells, whereas it has been reported that mice embryos lacking TF or TFR can still develop normally [29], suggesting that the alternative pathways for iron acquisition may exist. For example, ferritin not only functions during iron storage, but can also be secreted outside the cell to function as an iron donor [30]. During mouse embryonic kidney development, scavenger receptor class A member 5 (SCARA5) interacts with ferritin and promotes its endocytosis [31]. In human cells, TFR1 also interacts with ferritin and promotes its endocytosis [32,33]. In addition, it has been found that Zip14 is also responsible for the transportation of non-transferrin-bound iron (NTBI) into mouse hepatocytes and pancreatic cells [34]. A homologue of ZIP14, ZIP8, functions as an NTBI transporter in human proximal renal epithelial cells [35]. In sum, cellular iron uptake is a complex process and requires multiple molecular synergies. Therefore, further in-depth studies are essential for exploring the mechanism of iron uptake.

### 2.3. Cellular Iron Transfer to Mitochondria and Its Utilization 

Upon entering the cytoplasm, ferrous iron is directed towards the labile iron pool (LIP) [36], which is predominantly utilized by the mitochondria for the synthesis of Fe-S cluster proteins and hemoglobin. To avoid the redox toxicity of LIP, the remaining iron combines with iron chaperones after being released from endosomes via DMT1 [37], and then transfers to ferritin for storage (see below). For example, Vyoral et al. identified a high-molecular-weight intermediate in K562 cells incubated with TF, which appeared to transfer iron from chaperones to ferritin [38]. Furthermore, Shi et al. have shown that poly rC binding protein 1 (PCBP1) also acts as an iron molecular chaperone in yeast cells and Huh7 cells, donating iron to ferritin [39]. Recently, Wang et al. observed a decrease in iron and ferritin levels in the intestinal epithelium of PCBP1-deficient mice, indicating that PCBP1 is also crucial in regulating intestinal iron absorption and maintaining iron homeostasis in the body [40]. Except for transporting iron from the LIP to mitochondria, endosomes containing TF can also directly and temporarily attach to mitochondria to transport iron into them, bypassing the LIP [36]. This idea has been further developed into the “kiss and run” hypothesis [41,42]. Studies have demonstrated that myosin Vb [43], the cytoskeletal regulatory molecule MRCKα [44], and vesicle docking [45,46] all play a role in “kiss and run” hypothesis in in vivo and in vitro experiments. However, the exact mechanism of endosomal and mitochondrial contact is currently unknown. As iron enters the mitochondria, both outer and inner membranes must be crossed to reach the matrix for Fe-S cluster assembly and heme biosynthesis. DMT1 may be involved in iron crossing the outer mitochondrial membrane [47], whereas in vitro experiments clarified that iron flux through the inner mitochondrial membrane is dependent on mitoferrins 1 and 2 (MFRN1 and MFRN2) [48]. MFRN1 and MFRN2 are differentially expressed in tissues. MFRN1 is only highly expressed in erythrocytes whereas MFRN2 is widely expressed in mammalian tissues [49,50]. 

Within the mitochondria, iron normally has three destinations: the synthesis of hemoglobin [51], the synthesis of Fe-S clusters [52], and storage in mitochondrial ferritin [53]. The multiple steps of heme and Fe-S cluster synthesis occur in mitochondria and cytoplasm. In addition, there is a crosstalk between Fe-S clusters and heme. For example, a Fe-S cluster protein, ferrochelatase (FECH) catalyzes ferrous iron insertion into protoporphyrin (PPIX) macrocycles, which is the last step of heme synthesis [54]. 

### 2.4. Cellular Iron Storage

Iron that is temporarily unavailable to cells is stored in ferritin in cytoplasm and mitochondria. The ferritin molecule is composed of a spherical protein shell and an iron-containing core [55]. The protein shell consists of 24 polypeptide chains of different ratios of H and L subunits and its inner surface forms multiple contact cores with up to 4500 ferric iron [56]. The H subunit has redox activity and can catalyze ferrous iron oxidation to storable ferric iron [57], while the L subunits stabilizes the ferritin structure and store ferric iron by mineralization [58]. The ratio of H and L subunits can be varied in different cells, which determines the rate of metal mineralization [59,60]. When required, iron-containing ferritin is degraded by nuclear receptor coactivator 4 (NCOA4)-dependent autophagy (ferritin autophagy), allowing iron to flow back into the LIP [61]. Iron is stored in mitochondrial ferritin in mitochondria. Mitochondrial ferritin, like ferritin in the cytoplasm, has redox activity [53]. However, unlike cytoplasm ferritin, the expression of mitochondrial ferritin varies in different tissues. This suggests that the function of mitochondrial ferritin may be distinct from that of ferritin in cytoplasm.

### 2.5. Cellular Iron Export

Cellular iron export is a tightly regulated process that maintains intra- and extracellular iron homeostasis. Iron export is mainly divided into two pathways: FPN-dependent and independent pathways. FPN is characterized by two 6-membrane-helix bundles to form a channel for iron exportation [62,63]. Upon acceptance of Fe(II) carried by PCBP2, FPN undergoes a conformational change, leading to a channel opening to delivery of iron outside the cell [64]. To determine the critical role of FPN in iron absorption, it has been found that selective deletion of FPN from intestinal cells results in anemia in mice. In addition, knockout of FPN in macrophages and hepatocytes also developed anemia in mice fed with a low-iron diet but the mice fed with a normal diet had normal erythropoiesis. This result suggests that normal iron absorption can compensate for impaired iron recycling and iron release from storage tissues [65,66]. Aside from transferring iron to plasma, a study in a mouse model showed that FPN is also capable of releasing labile toxic iron from hemoglobin molecules that have been damaged [67]. 

Although FPN is the main factor for cellular iron export, other factors are also involved in this process. As mentioned above, in addition to functioning as an important molecule for iron storage, FPN also exists in the extracellular space as a carrier of iron ions [68]. Iron-containing ferritin can be secreted by ferritinophagy [30,69] or endosomal microautophagy [70,71] by membrane fusion. Ferritin secretion in exosomes can be stimulated by ferroptosis, iron overload and high lipid activity [68,70,72]. Iron can also be excreted from cells as heme iron. Feline leukemia virus subgroup C cellular receptor 1 (FLVCR1) on the cell membrane is responsible for heme transport [73], and mediates mouse erythroid differentiation [74]. The mutations of FLVCR1 cause abnormal oxidative stress responses in sensory neurons, resulting in sensory nerve-related diseases [75]. 

### 2.6. The Regulatory Mechanisms of Iron Hemostasis

#### 2.6.1. The Iron-Regulatory Protein (IRP) and Iron-Responsive Element (IRE) Regulatory System

Iron hemostasis can be regulated post-transcription. Iron-regulatory protein 1/2 (IRP1/2) can bind with cis-regulatory iron-responsive elements (IREs), which are specific stem-loop structures within the untranslated regions (UTRs) of the messenger RNAs (mRNAs) that encodes a variety of iron-related proteins [76]. The combination of IRP1 and IREs is regulated by the concentration and cellular need of iron. When iron concentration is low, IRP1 can bind with the 5′ UTR of the ferritin and FPN mRNAs to block their translation and bind with the 3′ UTR of the TFR1 mRNA to protect it from degradation by endoribonuclease regnases [77,78,79]. Conversely, when cellular iron concentration is high, IRP1 disassociates from IREs, thereby allowing ferritin and FPN for translation and exposing the TFR1 mRNA to degradation. Thus, the activation of the IRP-IRE system allows cells to have lower iron storage and export, but higher iron absorption. IRP2 regulation is crucial for embryonic development and hematopoiesis [80]. In addition to ferritin, FPN and TFR, the mRNAs of other iron-related proteins, such as DMT1 and HIF-2α, also contain IRE structures [76]. The IRP-IRE system is important for embryonic development, intestinal and liver function, immune function of macrophages and T-cells, erythropoiesis and iron management, and the development of neurological disorders [81,82,83,84,85,86]. 

#### 2.6.2. The Hypoxia-Inducible Factor (HIF) Regulatory System

HIF plays a crucial role in intestinal iron absorption. HIF is a heterodimeric nuclear transcription factor for many iron metabolism-related genes, such as TFRC (encoding TFR), SLC11A2 (encoding DMT1), etc. HIF consists of an oxygen-sensitive α subunit (HIF-1α/HIF-2α) and a common expressed β subunit (HIF-1β) [87,88,89]. Under normal oxygen conditions, two proline residues on HIF-1α and HIF-2α are hydroxylated by iron-dependent prolyl hydroxylase (PHD) and then recognized and degraded by the ubiquitin-proteasome system [90]. The functional integrity of PHD requires the cofactors ferrous iron and oxygen. Hence, under hypoxic or iron-deficiency conditions, the inactivation of PHD causes HIF-1/2α stabilization and combination with HIF-1β. Then, the complex is translocated into the nucleus, in which the complex binds to hypoxia response elements (HREs) to promote the transcription of hypoxia-inducible genes, including EPO, VEGF and GAPDH, to enhance angiogenesis and glycolysis process [91,92,93]. Of note, although HIF-2 overlaps with HIF-1 in some functions (e.g., regulating VEGF and EPO expression), HIF-2 plays a bigger role in iron metabolism. HIF-2 can directly bind to the promoter regions of the TFR, DCYTB and DMT1 genes, thereby promoting their transcription under hypoxic or iron-deficient conditions [94,95]. In addition, there is feedback between HIF-2 and the IRP-IRE regulatory pathway, in that the IRE region of HIF-2α can be bound by IRP to inhibit HIF-2α expression [96,97].

#### 2.6.3. Hepcidin–Ferroportin Regulatory System

**Figure 1:**
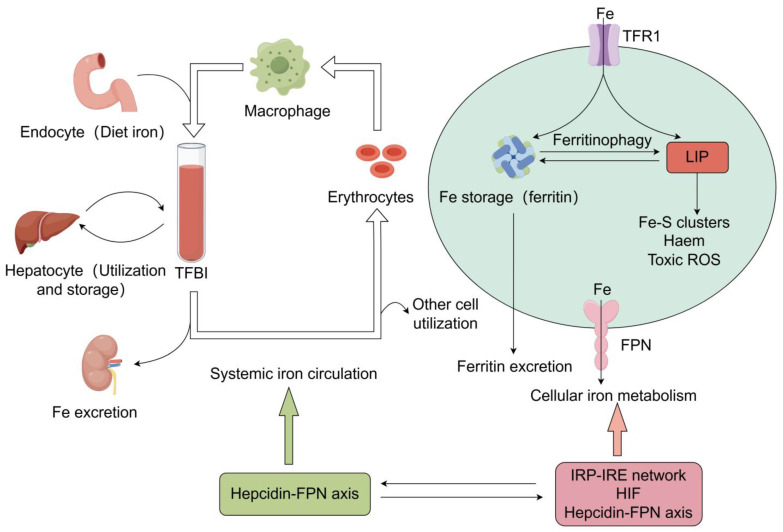
The regulation and the association between systemic iron circulation and cellular iron metabolism. Systemic and cellular iron are tightly regulated. Dietary iron is the only source of exogenous iron used to replace iron lost through iron excretion or bleeding. Dietary iron is absorbed into the bloodstream by enterocytes and exists in the circulation mainly as TFBI, which is recognized and taken up for use by cells of various tissues via TFR1. The liver is primarily responsible for systemic iron storage and regulates systemic iron homeostasis by synthesizing hepcidin. In the plasma, macrophages phagocytose senescent erythrocytes, releasing iron from hemoglobin and re-entering the circulation. Intracellularly, iron in the LIP is available for the synthesis of substances, such as the synthesis of heme by red lineage cells and the synthesis of Fe-S clusters by cells of various tissues, while excess iron is stored in ferritin. Ferritin can release the stored iron into the LIP via ferritinophagy, and intracellular iron can also be excreted directly from the cell via the FPN or by secretion of ferritin. When the intracellular iron regulation mechanism fails or the cellular iron is overloaded, the iron in LIP can generate toxic ROS through the Fenton reaction, leading to oxidative stress and cellular damage. Abbreviation: TFBI: transferrin bound iron, FPN: ferroportin, LIP: labile iron pool, HIF: hypoxia-inducible factor, IRP-IRE: iron-regulatory protein and iron-responsive element.

It is now known that FPN is highly expressed on the membrane surface of macrophages, enterocytes and hepatocytes, all of which are involved in increased iron fluxes. In contrast, the key iron-regulating hormone hepcidin (HAMP or HEPC), produced by hepatocytes, negatively feedback-regulates plasma membrane FPN levels [98,99]. Hepcidin modifies FPN through post-translational modification, leading to its ubiquitination, endocytosis and degradation by lysosomes [100,101]. Elevated iron levels in the circulation stimulate hepcidin expression, which is mediated by iron-sensing proteins (e.g., HFE, TFR2, HJV and BMP6) that sense changes in iron levels and promote the transcription of the hepcidin gene (HAMP) through the SMAD pathway [102,103,104]. The regulation between systemic iron circulation and cellular iron metabolism is shown in Figure 1. 

## 3. Renal Iron Homeostasis and Cellular Dysfunction in Kidney Diseases

### 3.1. Renal Iron Homeostasis

In addition to the universal iron transport proteins and regulatory pathways described above, there are kidney-specific iron uptake mechanisms. Due to different expressions of iron transport-related proteins, the ability to process iron varies greatly between different renal segments [105,106]. Under physiological conditions, proximal tubule epithelial cells can take up transferrin-bound iron (TFBI) from the apical membrane via TFR1, or via megalin-dependent and cubilin-mediated endocytosis [107,108]. The expression of TFR1 and megalin–cubilin complex is negatively associated in proximal tubules. Under the iron overloaded condition, the expression of TFR1 is decreased by the IRP-IRE in the kidney, whereas the expression of megalin receptor complex is increased in mouse proximal tubules [109]. In contrast, under iron deficient conditions, such as iron chelation, the expression of TFR1 is increased and the expression of the megalin–cubilin complex is decreased [110]. These results suggest a possibly converse effect between TFR1 and megalin–cubulin. Hemolytic conditions cause the mammalian proximal tubule to overburden with hemoglobin, resulting in the participation of both megalin and the neutrophil gelatinase-associated lipocalin receptor (NGALR) in the uptake of the filtered hemoglobin [111,112]. The level of ferritin and the abundance and activity of IRP1 are also higher in proximal tubules than in distal tubules, indicating that proximal tubules rather than distal tubules are primarily involved in cellular iron flux and renal iron reabsorption [108]. Furthermore, ZIP8, ZIP14 and DMT1 are expressed in the proximal and distal tubules in human kidney biopsy samples [113]. Given that ZIP8 and/or ZIP14 function at a higher pH than DMT1 for maximum iron transport, we can speculate that ZIP8 and/or ZIP14 may facilitate apical uptake of NTBI in mouse proximal tubules, while DMT1 may participate in subcellular uptake of apical NTBI [28,114,115]. In addition to the proximal tubule, the thick ascending limb of Henle’s loop, the distal tubule and collecting duct can also reabsorb TFBI for the expression of TFR1 and NGALR on the apical part of the cell [108,116]. 

Apart from the tubule’s reabsorption, glomerular epithelial cells also have an iron reabsorption function because of the expression of TFR1, FPN and DMT1 on cultured human glomerular epithelial cells [117]. Alfonso et al. show that human podocytes can take up hemoglobin through macrophage-mediated autophagy and metabolize it via heme oxygenase 1 (HO-1) degradation [118]. 

### 3.2. Abnormal Iron Metabolism and Cellular Dysfunction in Renal Injury and Disease

Iron homeostasis plays a vital role in maintaining normal organism function. Disrupted iron homeostasis can significantly impact various organs through both systemic iron overload and iron deficiency. Systemic iron overload leads to elevated plasma TFBI and non-transferrin bound iron (NTBI) levels and increased intracellular LIP, causing structural alternations of diverse proteins, and DNA due to the high redox capacity of iron [119]. An increased urinary iron excretion and renal iron deposition has been found in hemochromatosis (HH) mice [120]. Clinical case reports also indicate that HH can lead to hemosiderin deposition in kidneys and injured kidneys [121,122]. Hemochromatosis is an inherited disorder most commonly caused by mutations in the HFE gene that result in misfolding of the HFE protein, loss of function in sensing iron concentration and defective hepcidin synthesis in hepatocytes, which in turn leads to reduced ubiquitinated degradation of FPN, elevated FPN levels and increased release of cellular iron, resulting in elevated circulating iron levels [123]. In addition, hepcidin deficiency leads to an elevation of plasma TF saturation, inducing the accumulation of toxic NTBI in plasma [124]. Prolonged exposure of the kidney to enhanced levels of non-heme iron was found to potentially lead to renal injury in cultured porcine kidney epithelial cells [125]. 

Elevated renal injury markers were additionally observed in infants and children with iron deficiency anemia and could be corrected with oral iron therapy [126]. These findings suggest that an iron deficit may cause kidney damage and has a deleterious effect on renal function. In addition, patients with a variety of glomerular and tubular disorders, such as diabetic nephropathy and Fanconi syndrome, also have abnormal content of iron and TF in their urine [127,128,129,130,131]. In addition, patients with glomerular disease often exhibit hypo-transferrinogenemia due to the inability to adequately synthesize TF through the liver. However, there is little clinical evidence regarding whether iron deficiency impairs renal function.

## 4. The Regulation of Redox Homeostasis in the Kidneys

### 4.1. ROS and Oxidative Stress in the Kidneys

ROS can be categorized into two major classes of free radical- and non-free radical-oxidizing molecules (Table 1). Free radicals are molecules or atoms with unpaired electrons that are highly oxidizable and can directly react with intracellular biomolecules (e.g., proteins, lipids, DNA) in oxidative reactions, leading to cellular damage and oxidative stress [132]. Non-free radical ROS are oxidizing molecules that do not carry unpaired electrons and are less reactive compared to free radical ROS, but they can be converted to free radicals or participate in other oxidative reactions that can also cause cellular damage. ROS act in both directions, either as the “culprits” of oxidative stress, destroying cellular structures and causing damage (see Section 5), or as cellular signaling molecule that regulates various physiological processes. For example, hydrogen peroxide participates in the activation of the epidermal growth factor receptor (EGFR), which in turn activates a number of downstream signaling pathways and promotes cell proliferation and survival [133,134,135]. Mitochondrial ROS also play an important role in T cell activation, and inhibition of mitochondrial ROS production can reduce T cell activation in vivo or in vitro experiments [136]. Recent studies have shown that mitochondrial ROS also contribute to the bactericidal activity of macrophages [137]. In addition, ROS play an important role in the regulation of cellular senescence and stem cell differentiation, which will not be discussed in this article.

The primary producers of endogenous ROS in the kidneys of mammals are mitochondria and NADPH oxidases (NOX) [138,139,140]. As kidneys require large amounts of energy (ATP) for filtration and reabsorption, mitochondria serve as the main source of cellular ATP to power these activities [141]. Mammalian mitochondria can produce superoxide (O_2_^−^) and/or hydrogen peroxide through substrate catabolism and electron leakage in the electron transfer chain (ETC). During electron transfer, electrons may escape into oxygen to yield O_2_^−^ and/or hydrogen peroxide. As the substrate of Fenton reaction, hydrogen peroxide can react with ferrous iron from LIP and produce hydroxyl radicals, a ROS with strong oxidization activity [142]. Previous studies suggest ROS are mainly produced from ETC complexes I and III, while mitochondrial pyruvate dehydrogenase and α-ketoglutarate dehydrogenase are involved in this process [143]. Most mitochondrial enzymes near ROS production centers contain thiol groups modified by ROS, implying that mitochondrial ROS production is tightly self-regulated [144]. The NOX family contains seven homologous isoforms differentially distributed among tissues, in which NOX4 is predominant in kidneys [145]. NOX4 is localized mainly to mitochondria, the endoplasmic reticulum and plasma membrane [145,146]. Due to its unique E-loop structure, NOX4 can produce hydrogen peroxide rather than superoxide [147].

Stimulated by oxidative stress, NO synthase (NOS) can be converted from NO-producing enzymes to superoxide-producing enzymes, a process that is known as NOS uncoupling [148]. Uncoupling of NOS leads to NOS dysfunction and contributes to ROS overproduction. Three types of NOS exist in the body: neuronal NOS (nNOS, also called NOS1), inducible NOS (iNOS, also called NOS2) and endothelial NOS (eNOS, also called NOS3). nNOS and eNOS are constitutively expressed, whereas iNOS expression associates with inflammation [148]. It has been found that nNOS is expressed in cortical tubules and eNOS is expressed in glomeruli [149]. NOS mainly mediates endogenous NO synthesis [149]. NO can induce cGMP production, a second messenger which affects cardiovascular, renal and metabolic systems beneficially. However, NOS uncoupling increases ROS production and decreases NO bioavailability, which exacerbates CKD [149,150]. A key eNOS function is to regulate blood flow and maintain endothelial integrity. Mice lacking eNOS exhibited symptoms similar to metabolic syndrome and developed severe kidney disease [151,152]; while supplementing nitrate in the diet could reverse the disease outcome [153]. In addition, eNOS uncoupling and NOX activation were found to generate ROS, leading to endothelial dysfunction in DKD rats [154,155]. When ROS accumulate and cells undergo oxidative stress, the eNOS coenzyme tetrahydrobiopterin (BH4) oxidizes to BH2 by ROS, inducing eNOS uncoupling [156]. At this point, NOS becomes unstable, transferring electrons from L-arginine that should fuel eNOS to oxygen instead, producing O_2_^−^ [157]. Treatment with BH4 prevents eNOS uncoupling, reduces ROS and improves endothelial function [158,159]. Patients treated with BH4 in clinics showed decreased urinary albumin excretion [154,160].

### 4.2. Antioxidant Defense Systems in the Kidneys

Like most tissues and organs, the kidney contains an enzymatic and a non-enzymatic antioxidant system (Table 2). The enzymatic system consists of superoxide dismutase (SOD), catalase (CAT), glutathione peroxidase (GPX) and thioredoxin (Trx). The non-enzymatic system is mainly composed of reduced glutathione (GSH) and other molecules. 

SOD catalyzes the transformation of superoxide anion into oxygen and hydrogen peroxide, mitigating the effects of ROS. The activity of SOD in mammalian kidneys is largely mediated by SOD1, and reduction of SOD1 has been associated with kidney injury [161,162]. CAT is localized in peroxisomes, which are highly expressed in the liver, lungs and kidneys and reduce SOD-generated hydrogen peroxide to oxygen and water [163]. Antioxidant enzymes like CAT, SOD1 and PRX are found in peroxisomes, which are organelles that control the oxidative state of cells [164]. Targeted phosphorylation of the peroxisomal membrane protein PEX14 shields DNA from ROS by elevating cytoplasmic levels of CAT during mitotic nuclear membrane dissolution in mammalian cells [165]. However, peroxisomes are also involved in fatty acid breakdown through β-oxidation, which produces hydrogen peroxide as a byproduct [166]. 

GPX can function similarly to CAT to reduce SOD-derived hydrogen peroxide to oxygen and water [167,168]. In addition, GPX specifically reduces lipid peroxides and prevents the lipid peroxidation chain reaction, thereby preventing cell membrane disruption and cell necrosis [167]. Of these, GPX4 is the only known intracellular enzyme that is resistant to phospholipid peroxidation, and GPX4 deficiency is associated with ferroptosis through increased ROS, lipid peroxidation and iron overload [169,170].

Trx primarily accomplishes its antioxidant role by passing electrons to Trx-dependent peroxidases (Prxs), methionine sulfoxide reductases (Msr) and other oxidation-sensitive molecules [171,172]. The Trx system can transfer electrons to Prxs and Msr to remove ROS [173]. Trx then restores redox activity by receiving electrons from TrxR [174]. NADPH, in turn, can transfer electrons to Trx reductase (TrxR) via FAD [175,176]. Furthermore, it was found that GSH can reduce oxidized Trx in vitro, suggesting that there may be crosstalk between the Trx system and GSH [177].

GSH is involved in the scavenging of peroxides as a cofactor of GPX [178,179]. In addition, GSH can reduce two oxidation products, protein thiyl radicals and protein thiolate, back to protein sulfhydryl groups (protein-SH) [180]. GSH can also restore the free radicals of other antioxidants that have lost their antioxidant capacity, such as vitamins C and E, back to their functional, non-free radical state [181].

## 5. Oxidative Stress and Cellular Dysfunction in Kidney Diseases

Physiological concentration of ROS makes them function as signaling molecules that control a number of critical physiological processes [182]. However, overproduction of ROS disrupts redox homeostasis, leading to oxidative damage to important biomolecules such as DNA, proteins and lipids [183].

### 5.1. Oxidative Stress and Renal Tubular Cell Dysfunction in Kidney Disease

It is generally estimated that each human cell is exposed to about 10^5^ attacks per day from ROS such as hydroxyl radicals [184]. Damage to DNA by ROS identified so far includes bases modification, deletions, translocations, DNA strand breaks and DNA-protein cross-links and chromosomal rearrangements [185]. Hydroxyl radicals, which can react with all of DNA’s constituents, including the deoxyribose backbone and purine and pyrimidine bases, are primarily responsible for DNA damage [186,187]. These injuries lead to genetic variations, such as mutations and chromosomal rearrangements, which may lead to cell senescence [188]. Renal tubular epithelial cells are commonly associated with renal senescence. Increased tubular epithelial cell senescence was found in animal models of AKI (IRI) and CKD (UUO) as well as in CKD patients [189,190]. Senescent cells are able to secrete various cytokines, such as IL-1β, IL-6, IL-8 and TGFβ1, collectively known as the senescence-associated secretory phenotype (SASP), which significantly affects neighboring cell and tissue functions [191,192,193]. SASP mediates inflammatory and pro-fibrotic responses, which activate renal interstitial fibroblasts, contributing to an overproduction of matrix proteins, which disrupts the physiological structure of renal tissues and promotes the formation of fibrous scars [194].

Apoptosis has been observed in human renal tubular cells of diabetic patients due to high glucose-induced overproduction of ROS and disruption of mitochondrial structure, which leads to decreased mitochondrial respiratory function and ATP production, thus causing energy metabolism disorders and renal tubular cells’ apoptosis [195,196]. In addition, the upregulation of kidney injury molecule-1 (KIM-1) in mouse renal tubular epithelial cells during the course of DKD led to an increase of the internalization of palmitic acid albumin by proximal tubular cells and an activation of NLRP3 inflammatory vesicles by palmitic acid via ROS production, leading to the production of pro-inflammatory factors, such as IL-1β, thereby inducing renal tubular cell death, mitochondrial fragmentation and subsequent fibrosis [197,198,199].

Acute tubular injury is the most frequent pathologic manifestation of AKI. The etiology of prerenal AKI is inadequate perfusion due to decreased renal blood flow, which usually manifests as acute ischemic tubular damage [200]. Under ischemia and hypoxia, renal tubular cells, which are highly metabolically active, are severely injured due to their high oxygen demand [201]. The most common initial manifestations of ischemic AKI are ATP reduction and the alterations of mitochondrial structures [202]. The conditions of hypoxia and ischemia can induce oxidative stress, which leads to strong oxidative damage to mitochondrial lipids and proteins, disrupting energetic metabolism via disrupting ETC function [203]. According to multiphoton imaging reports, the mitochondrion is the major source of ROS in a mouse ischemic AKI model [203]. Subsequently, post-injury renal tubular epithelial cells undergo cell death, such as apoptosis and necrosis, releasing inflammatory mediators that in turn exacerbate tubular injury [204]. In addition, reperfusion after ischemia also causes injury due to mitochondrial ROS bursts [205,206], which can induce infiltration of inflammatory cells, such as neutrophils, early in IRI. ROS and inflammatory factors originating from neutrophils can further aggravate renal tubular injury in rats [207,208]. Furthermore, uncoupling protein 1 (UCP1) in renal tubular epithelial cells is downregulated in a time-dependent manner during renal ischemia–reperfusion, resulting in elevated renal oxidative stress and exacerbating ischemia-induced AKI in mice [209].

ROS are also involved in several aspects of renal tubular cell injury in septic AKI (SAKI). Pathogen invasion activates the innate immune system, and neutrophils and macrophages protect the organism with oxidants such as hydroxyl radicals and peroxides [210]. The generation of ROS during defense can activate the inflammatory response to increased iNOS, resulting in excess NO, which in turn leads to uncoupling of eNOS and production of highly reactive peroxynitrite radicals [211,212]. In addition, excess NO reacts with peroxo-anion radicals to generate peroxynitrite to directly damage renal tubular cells [213,214]. 

Nephrotoxic drugs such as antibiotics and chemotherapeutic drugs can directly damage kidney tubular cells, leading to AKI. For example, cisplatin increases ROS production and depletes antioxidant molecules such as GSH in vivo, leading to endogenous ROS accumulation in renal tubular cells and then cell death [215]. In addition, cisplatin may induce mitochondrial dysfunction, leading to increased mtROS production in microsomes via cytochrome P450 enzymes, further exacerbating oxidative stress injury in porcine kidneys [216].

### 5.2. Oxidative Stress and Podocyte Dysfunction in Kidney Diseases

Loss of podocytes is a feature of early diabetic nephropathy and is involved in the progression of DKD. Increased extracellular glucose has been found to stimulate intracellular ROS production via NOX and mitochondrial pathways, which leads to the activation of p38 mitogen-activated protein kinase and caspase 3, ultimately resulting in podocyte apoptosis [217]. Further studies revealed that Ras-related C3 botulinum toxin substrate 1 (Rac1) plays a role in podocyte injury by regulating multiple signaling pathways [218,219]. Rac1 can be activated by various stimuli, including ROS, inflammatory cytokines, angiotensin II and high glucose concentration [220,221,222]. The activation of Rac1 activates various downstream effector molecules and signaling proteins, such as WAVE complex, mDia2, PAK and LIM kinase, which are involved in actin cytoskeleton remodeling, leading to increased mouse podocyte motility [223]. Normal podocytes have a stationary phenotype, whereas damaged podocytes are transformed into a Rac-dependent motile phenotype. The motile phenotype is related to the increase of podocyte motility, loss of podocyte process and proteinuria [224]. Motile podocytes may eventually be depleted, leading to podocytopenia and focal segmental glomerulosclerosis (FSGS) [225]. In addition, angiotensin II activates the Rac1-NOX-ROS cascade reaction by binding to the type I angiotensin II receptor, leading to rearrangement of the actin cytoskeleton of podocytes and increased motility [226]. Similar to renal tubular epithelial cells, podocytes can internalize plasma proteins via free fatty acid receptor-mediated endocytosis, leading to protein accumulation in endoplasmic reticulum (ER) and dysregulation of unfolded protein reaction pathways, ultimately causing ER stress and mitochondrial damage [227,228]. ER stress is considered to be induced by ROS, whereas mitochondrial damage generates ROS, creating a vicious cycle that is detrimental to podocytes.

### 5.3. Oxidative Stress and Mesangial Cell and Interstitial Fibroblast Dysfunction in Kidney Disease

Glomerular mesangial cells and interstitial fibroblasts are thought to be involved in excessive extracellular matrix synthesis during the progression of CKD. Both cell types respond very similarly to pro-fibrotic stimuli during disease progression, such as by transforming to a myofibroblast phenotype upon activation and synthesizing matrix proteins, including fibronectin, laminin and collagen types I, III and IV [229,230,231,232,233]. It has been reported that a variety of stimuli including TGF-β, angiotensin II and hyperglycemia have been shown to alter the activity of NOX in both mesangial cells and fibroblasts, and ultimately the amount of ROS production [234,235]. More specifically, NOX4 is a major mediator of the activation of thylakoids and the conversion of fibroblasts to myofibroblasts caused by high glucose or angiotensin II [145,236,237]. Increased ROS synthesis in activated mesangial cells and myofibroblasts also activates downstream signaling pathways, leading to increased extracellular matrix synthesis and renal fibrosis.

### 5.4. Oxidative Stress and Endothelial Cell Dysfunction in Kidney Disease

As discussed above, endothelial cells’ main function is the synthesis and secretion of NO via eNOS. NO is involved in various life processes through cGMP-mediated vasodilation, inflammation and immune responses. Under normal conditions, low NO levels in the endothelium can induce antioxidant gene expression and inhibit cytochrome C oxidase, reducing ROS production [238]. However, elevated ROS levels reduce endothelial NO production by inhibiting and/or uncoupling NOS [239,240]. For example, in early CKD patients, plasma accumulation of ADMA (an L-arginine analogue) and uncoupling of NOS lead to inhibition of NO synthesis and increased ROS production [239,241]. Subsequently, renal endothelial dysfunction and increased vasoconstrictive blood flow resistance lead to glomerular ischemia. In addition, the reaction of NO with superoxide produces peroxynitrite, which further leads to tissue damage by reacting with thiols, lipids and proteins containing aromatic amino acids [241,242].

## 6. Oxidative Stress-Related Molecules in Kidney Disease

### 6.1. Nrf2

Nrf2 is a transcription factor that responds to intracellular oxidative stress and is involved in the regulation of cellular redox homeostasis. Normally, Nrf2 interacts with kelch-like ECH-associated protein 1 (KEAP1) to be sequestered in the cytoplasm. Subsequently, Nrf2 and E3 ubiquitin ligase are ligated, causing Nrf2 degradation via the proteasome [243]. During oxidative stress, ROS modify critical cysteine residues within KEAP1, inducing a conformational change that disrupts the KEAP1-Nrf2 interaction and allows Nrf2 to evade degradation [244]. Nrf2 then translocates to the nucleus and binds to antioxidant response elements (AREs) within promoter regions of various antioxidant and cytoprotective genes, including GSH S-transferases, HO-1, CAT, γ-glutamylcysteine synthetase and NAD(P)H quinone oxidoreductase, to increase their expression [245]. Nrf2 is also responsible for the regulation of GSH and Trx systems. Nrf2 regulates the expression of GSH synthases, such as glutamate–cysteine ligase (GCL) enzyme complex, and the amount of the limiting substrate of GSH, cysteine [246,247], to regulate GSH expression. Nrf2 also regulates the expression of Trx through the induction of NADPH, which is crucial for electron transfer in the Trx system [248].

The renoprotective functions of Nrf2 have been demonstrated in various CKD models, and serves to attenuate oxidative stress induced by pathological conditions, such as ischemia-reperfusion injury (IRI), unilateral ureteral obstruction (UUO), nephrotoxicants and diabetes mellitus (DM), to slow disease progression [249]. Nrf2 deficiency in a UUO mice model decreased the expression of antioxidant genes glutamate-cysteine ligase catalytic (Gclc) and HO-1 and increased the expression of genes associated with inflammation and fibrosis such as TGF-β, TNF, IL-6, IL-1b, etc., suggesting that the protective function of Nrf2 for renal tubulointerstitial fibrosis may be facilitated by both anti-inflammatory and antioxidant pathways [250]. DKD is a disease that develops and progresses due to a number of interconnected pathogenic mechanisms, such as altered renal metabolism, mitochondrial dysfunction and oxidative stress. Dandona P. et al. demonstrated that the levels of 8-hydroxy-2′-deoxyguanosine, a biomarker of oxidative stress, were elevated in the urine of diabetic patients relative to controls and correlated positively with other indicators of complications such as proteinuria [251]. Furthermore, in a streptozotocin-induced mice model of diabetes, knockout of Nrf2 led to more intense DNA damage and more ROS production, resulting in severe proteinuria and glomerulosclerosis compared to wild-type controls [252,253]. Nrf2 also confers protective effects on pancreatic beta cells and mitigates insulin resistance in individuals with diabetes [254]. In addition, Nrf2-deficient mice are more susceptible to cisplatin-induced kidney injury than wild-type mice [255]. Pretreatment of wild-type mice with the Nrf2 activator CDDO-Im prevents cisplatin-induced nephrotoxicity [256]. With the in-depth study of Nrf2 activators, a variety of compounds such as astragaloside IV, bardoxolone methy, etc., can attenuate cisplatin-induced renal injury via the activation of the Nrf2/HO-1 signaling pathway [257,258]. Nrf2 activators significantly improved ischemia/reperfusion- and LPS-induced AKI [259]. Qiu et al. have demonstrated that treatment with obacunone, an activator of the Nrf2 pathway, significantly decreased mouse renal cyst growth in autosomal dominant polycystic kidney disease (ADPKD) mice models by suppressing lipid peroxidation through upregulation of glutathione peroxidase 4 (GPX4), and inhibition of lipid peroxidation ultimately led to a decrease of hypercellular proliferation through decreasing the activation of the mechanistic target of rapamycin (mTOR) and mitogen-activated protein kinase (MAPK) signaling cascades [260]. 

### 6.2. NF-κB

Inflammation is a major contributor to CKD progression. The renal inflammatory microenvironment consists of inflammatory factors and immune cells. Macrophages, in particular, play a key role in renal inflammation [261]. Macrophages exhibit either M1 or M2 phenotypes. M1 macrophages secrete proinflammatory mediators like ROS, TNF-α, and IL-1β, thereby promoting inflammation and injury. Conversely, M2 macrophages exhibit an anti-inflammatory phenotype through IL-10 secretion, which counteracts acute inflammation caused by M1 cells [262]. However, sustained M2 responses can also result in excessive extracellular matrix (ECM) deposition and exacerbate renal fibrosis [263]. 

Nuclear factor kappa B (NF-κB) is a ubiquitous transcription factor that normally exists as an inactive cytoplasmic complex through combination with inhibitory κB (IκB) proteins [264]. The activation of the toll-like receptor by TNF-α and IL-1β induces the activation of the IκB kinase complex (IKK), leading to IκB phosphorylation and degradation [264]. This allows NF-κB translocation to the nuclear region, to increase the transcription of proinflammatory genes such as IL-1, IL-6 and TNF-α. Persistent NF-κB activation is associated with chronic inflammation in CKD [265,266]. ROS can activate NF-κB to drive renal fibrotic progression, while NF-κB also upregulates NOX and NOS to exacerbate oxidative stress [267,268]. Nrf2 can inhibit NF-κB signaling and decrease the expression of genes stimulated by NF-κB [245]. 

Renal oxidative stress and inflammation, which are symbols of DKD, are considered to be regulated by NF-κB. ROS can activate NF-κB signaling, thereby modulating the expression of various adhesion molecules, pro-inflammatory cytokines, and chemokines involved in chronic renal inflammation in DKD [269]. Prolonged activation of NF-κB-mediated inflammation exacerbates oxidative injury to renal cells. Additionally, oxidative stress and inflammation disrupt the normal balance between ECM synthesis and degradation, resulting in excessive ECM accumulation in the kidneys [270,271]. The cumulative effects of aberrant NF-κB signaling, oxidative stress and inflammation ultimately promote renal fibrosis, a key pathological feature of advanced DKD [272]. Administration of sappanone A (SA) mitigated systemic inflammation and cortical renal inflammatory responses as well as renal injury in a murine model of DKD by modulating the NF-κB signaling cascade [273].

It is well known that systemic inhibition of NF-κB can influence the severity of AKI. Inhibition of NF-κB attenuated kidney injury in a folic acid-induced AKI model [274]. Inhibition of IκB kinase at 24 h after the onset of AKI also effectively suppressed NF-κB activity, which in turn improved renal function and attenuated fibrosis [275]. Further studies found that NF-κB plays a role in AKI through immune cells and epithelial cells. Inhibition of NF-κB in macrophages by blocking CD38 can alleviate LPS-induced AKI in mice [276]. Additionally, NF-κB activation may augment M1 macrophage infiltration in AKI through the C-type lectin receptor Mincle [277]. In an ischemia/cisplatin-induced mice AKI model, Yang et al. proposed that KIM-1 mediates phagocytosis of apoptotic epithelial cells to attenuate the inflammatory response, which may be achieved by decreasing the activation of NF-κB [278]. Reduced apoptosis, lowered chemokine expression and attenuated AKI were also observed in mice with renal tubular epithelial cell-specific NF-κB knockout [279]. 

### 6.3. Sirtuin 1

The seven mammalian sirtuins (SIRT1-7) regulate various processes related to antioxidant capacity, oxidative stress and metabolism. SIRT1, the most extensively characterized isoform, localizes to the cytoplasm and nucleus [280]. As a NAD+-dependent deacetylase, SIRT1 helps maintain redox homeostasis by modulating the ratio of NAD+/NADH [281,282,283]. SIRT1 inhibits inflammatory responses and oxidative stress by deacetylating the p65 subunit of NF-κB [284]. In human renal tubular epithelial cells (HK-2) cultured with high glucose, SIRT1 deacetylation activity is decreased, resulting in a decrease of p65 deacetylation and then an increase of NF-κB activity, which decrease the expression of miR-29 through directly binding to its promoter. MiR-29 has an ability to target and suppress Keap1 gene expression. The decrease of miR-29 increases Keap1 expression and inhibits the Nrf2/ARE (antioxidant response element) pathway [285]. SIRT1 also promotes the activation of endothelial NOS in rats, leading to more NO production and preventing oxidative stress [286]. FOXO1 and FOXO3 regulate antioxidant genes like SOD2 and CAT through SIRT1-mediated deacetylation [287]. By modifying the transcription of the FoxO target gene, SIRT1 protects diabetic kidneys and blood vessels from oxidative stress and tissue damage [287]. In addition, SIRT1 inhibits p53-dependent apoptosis arising from DNA damage and oxidative stress through p53 deacetylation, promoting human renal cell survival [288]. SIRT1 also negatively regulates the mitochondrial ROS producer p66Shc by promoting its deacetylation, reducing oxidative stress and maintaining mammalian endothelial function [289]. 

The activation of SIRT1 prevents CKD by mechanisms such as increasing COX-2 expression and attenuating renal fibrosis and inflammation [290]. In a rat model of CKD, SIRT1 activation attenuated inflammation and tubular fibrosis via inhibition of the TGF-β/SMAD pathway [290,291,292]. In DKD, activation of SIRT1 confers renoprotective effects across various models of renal injury. SIRT1 predominantly exerts protection in proximal tubular cells and podocytes. Activation of SIRT1 can reduce the expression of the pro-apoptotic gene, Bcl2-like 11 (Bcl2l11), through deacetylation of FOXO4, thereby preventing podocyte loss in diabetics [293]. SIRT1 also inactivates NF-κB and the signal transducer and activator of transcription 3 (STAT3) to attenuate proteinuria and podocyte damage in db/db mice [294]. Increased proteinuria and progression of DKD were observed in a mouse model with podocyte-specific SIRT1 knockout [295]. In addition, Hasegawa et al. proposed that knockout of SIRT1 increased the expression of the tight junction protein claudin-1 in proximal renal tubules, exacerbating albuminuria and impairing renal function in in vivo and vitro experiments [296]. 

**Figure 2:**
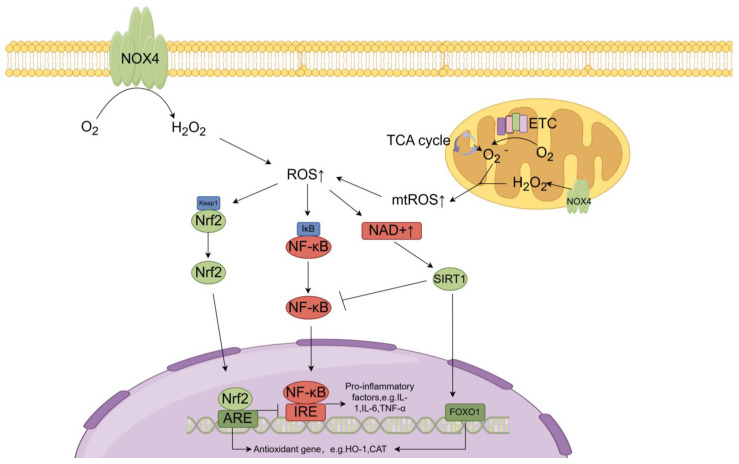
The main sources of ROS and ROS mediated the activation of Nrf2, NF-κB and SIRT1 in kidneys. Intracellular ROS in renal tissues is mainly derived from the escape of O_2_^−^ and H_2_O_2_ during mitochondrial aerobic respiratory electron transfer and NOX4 transfers electrons directly to oxygen molecules to produce H_2_O_2._ When ROS is elevated, the structure of Keap1-Nrf2 is disrupted, resulting in the release and entry of Nrf2 into the nucleus, where it binds to the ARE in DNA and increases the expression of downstream antioxidant genes. In addition, NF-κB is bound under normal conditions by IκB, which exists as an inactive complex in the cytoplasm. When intracellular ROS is elevated, IκB would be dissociated from NF-κB, leading to the activation of NF-κB activation and its translocation into the nucleus, where it binds to IRE in DNA and initiates the expression of downstream pro-inflammatory genes. Furthermore, elevated ROS can also lead to an increase of NAD+ and the activation of SIRT1, a NAD+-dependent deacetylase capable of inhibiting NF-κB activity by deacetylating its p65 subunit, thereby inhibiting the inflammatory response. In addition, SIRT1 promotes FOXO1 activation and nuclear translocation by deacetylating FOXO1, leading to an increase of the expression of various antioxidant genes, such as SOD2 and CAT. Abbreviation: NOX4: NADPH oxidases 4, ETC: electron transfer chain, Keap1: kelch-like ECH-associated protein 1, Nrf2: nuclear factor erythroid 2-related factor 2, ARE: antioxidant response element, IκB: inhibitory κB, NF-κB: nuclear factor kappa B, IRE: iron-responsive element, SIRT1: sirtuin 1, FOXO1: forkhead box O1, HO-1: heme oxygenase 1, CAT: catalase.

In a rat model of IRI-induced AKI, upregulation of SIRT1 effectively recovered renal function and attenuated apoptosis compared to controls. SIRT1 expression is observed to increase in HK-2 cells cultured under hypoxic conditions to reduce ROS production [297] (Figure 2).

## 7. The Crosstalk of Abnormal Iron Metabolism and Oxidative Stress in Kidney Diseases

### 7.1. How Oxidative Stress Affects Iron Metabolism

It has been found that ROS can alter the activity of Fe-S clusters in IRP1 and influences the activity of IRP1 binding with mRNA and subsequent cellular iron metabolism [298,299,300]. Intracellular superoxide anion inhibits cellular iron uptake capacity by inactivating IRP1, resulting in its inability to bind to the IRE segment of mRNA [301]. Treatment with exogenous hydrogen peroxide stimulates Fe-S clusters’ catabolism, which promotes IRP1 binding to mRNA and increases iron endocytosis in cultured mouse fibroblasts cells [302]. In addition, an IRP-IRE-independent mechanism of iron regulation was identified. The transcriptional level of cellular TFR was elevated upon treatment with low concentrations of hydrogen peroxide, and the ability of IRP to bind to mRNA was unchanged [303]. In contrast, treatment with high concentrations of hydrogen peroxide increased ferritin proteasome degradation in microglial cells [304]. In addition, treatment with hydrogen peroxide may rapidly inactivate PHD by oxidizing divalent iron in the PHD active site and lead to transient activation of HIF-1α in human osteosarcoma cells. However, prolonged hydrogen peroxide treatment results in the activation of ferrireductase and restores enzymatic activity of PHD, resulting in a decrease in HIF-1α [305].

### 7.2. Abnormal Iron Metabolism Leads to Oxidative Stress and Ferroptosis

**Figure 3:**
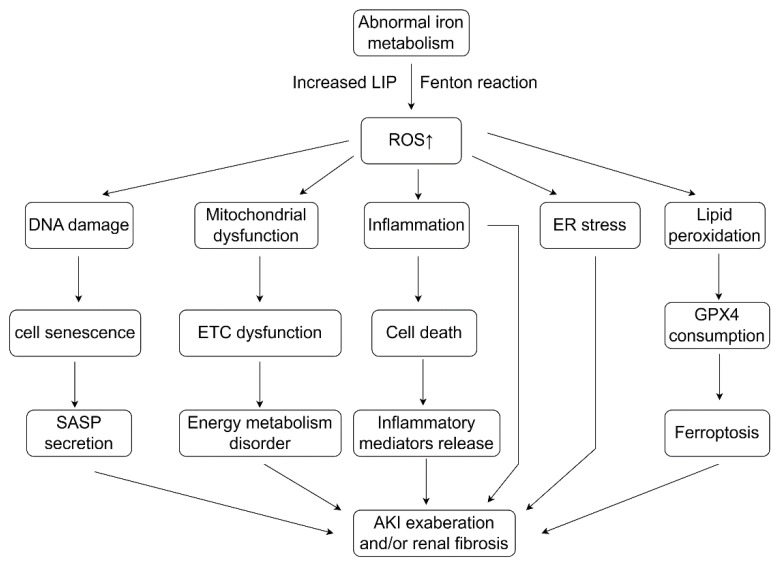
Oxidative stress as a mediating factor connecting abnormal iron metabolism and aggravation of kidney diseases. Abnormal iron metabolism leads to oxidative stress by generating excess ROS through the Fenton reaction. Increased ROS promotes DNA damage, mitochondrial dysfunction, inflammatory response, ER stress and lipid peroxidation (ferroptosis), resulting in an increase of cellular senescence, cell death, ETC dysfunction and ferroptosis. Subsequently, this causes irreversible renal damage because of loss of irreducible cells, such as podocytes and endothelial cells, as well as abnormal tissue repair (nonfunctional scar repair), leading to the increase of the progression of renal disease to further deteriorate renal function. Abbreviation: SASP: senescence-associated secretory phenotype, ER: endoplasmic reticulum, GPX4: glutathione peroxidase 4, AKI: acute kidney disease.

In patients with CKD, intracellular iron efflux is usually blocked, leading to intracellular accumulation of iron. Elevated serum hepcidin levels have been found in patients with CKD, which subsequently binds to FPN on the cell membrane and leads to FPN degradation and intracellular iron accumulation [306,307]. Excess iron subsequently produces hydroxyl radicals via the Fenton reaction, which can react with PUFAs on cell membranes and oxygen to form lipid ROS or lipid peroxyl radicals. This results in ferroptosis, a genetically and biochemically unique kind of programmed cell death that is different from others [308]. Ferroptosis is an iron-dependent programmed cell death that is characterized by the accumulation of lipid peroxides and regulates the progression of certain renal disorders [309] (Figure 3).

Recent studies have indicated that ferroptosis significantly leads to the pathogenesis of renal tubular injury progressing to renal fibrosis [310]. Recently, it has been found that repressor element 1-silencing transcription factor (REST) is upregulated in renal tubular epithelial cells of AKI patients and mice and leads to renal injury through the regulation of ferroptosis, and that tubular-specific knockdown of REST significantly attenuates the transition from AKI to CKD and ameliorates renal fibrosis [311]. Treatment with ferroptosis inducers, such as erastin, decreases GPX 4 activity and enhances intracellular lipid peroxidation to aggravate renal fibrosis [312]. Pretreatment with ferroptosis inhibitors stopped the progression of renal fibrosis by preventing ferroptosis-related lipid peroxidation and GSH depletion. In addition, vitexin attenuates CKD by inhibiting ferroptosis in renal tubular epithelial cells through activation of Nrf2 [312], and formononetin and tectorigenin attenuate renal fibrosis by inhibiting Smad3 [313,314].

Iron-dependent lipid peroxidation also exists in db/db and STZ-induced mouse or rat models of DKD and in DKD patients [315,316,317,318]. It has been found that under the condition of high glucose, HK-2 cells were characterized with an iron overload, a reduction of antioxidant ability, an accumulation of ROS and a lipid peroxidation [315,319], whereas all of these could be alleviated by the treatment with ferrostatin-1 (Fer-1), a ferroptosis inhibitor2, suggesting a role of ferroptosis in DKD [315,319]. ZIP14, a cellular iron importer, was upregulated while GPX4 and GSH levels were decreased in rat DKD models and Fer-1 treatment could normalize iron and ZIP14 levels [318]. Increasing mitochondrial GSH content with N-acetylcysteine (NAC) also significantly ameliorated high glucose-induced ferroptosis in renal cells [320]. 

Ferroptosis also participates in the process of ADPKD [321]. Schreiber et al. discovered that in the renal tissues of ADPKD patients as well as in a mouse model, lipid peroxidation enhanced the activation of the chloride channel TMEM16A, which is closely linked to renal cyst enlargement [322]. In contrast, treatment with Fer-1 effectively inhibited TMEM16A activation and cyst enlargement. Mutations of polycystin 1 predisposed cells to ferroptosis by dysregulating iron and lipid metabolism [323]. In addition, high iron levels, low GPX 4 activity and increased lipid peroxide accumulation were verified in both ADPKD cells and mouse models [323].

A kidney biopsy report of a patient with IgA nephropathy shows a significant deposition of iron-containing hemosiderin in the renal tubules [324]. A cohort study revealed that compared to the healthy control group, IgA patients had significantly lower levels of the antioxidant enzymes SOD and vitamin E in their serum, while the levels of the intermediate product of lipid peroxidation, malondialdehyde (MDA), were elevated [325]. Furthermore, Wu et al. confirmed that GPX4 levels were significantly decreased in kidneys of IgA patients, and treatment with the ferroptosis inhibitor Fer-1 resulted in a decrease of MDA and ROS, an increase of GPX4, an inhibition of ferroptosis in mesangial cells and a delay of the progression of IgA nephropathy [326]. These findings suggest that ferroptosis plays a crucial promoting role in the progression of IgA nephropathy.

In the mouse IRI model of AKI, single-cell sequencing showed that ferroptosis-related genes are predominantly expressed in the renal tubular epithelial cells [327]. Conditional knockout of ferroptosis suppressor protein 1 (FSP1) or GPX4 increased cellular sensitivity to iron death and exacerbated acute tubular necrosis in a mouse model of IRI-AKI [328]. Another study found that knockout of ACSL4 (a promoter of ferroptosis) effectively reduced pathological injury in AKI mice [329]. In addition, treatment with ferroptosis inhibitors has been shown to effectively alleviate IRI-AKI in mice model [327,330]. Ferroptosis is also increased in sepsis-induced AKI mouse models [331]. Recent studies have demonstrated that treatment with melatonin prevents ferroptosis and ameliorates sepsis-associated AKI by upregulating Nrf2/HO-1 cytoprotective pathway [332]. Furthermore, ferroptosis is also involved in the injury response in rhabdomyolysis and cisplatin-induced AKI [333,334]

## 8. Conclusions and Perspectives

Abnormal iron metabolism and oxidative stress play crucial roles in the progression of kidney diseases, ultimately resulting in end-stage renal disease. Iron homeostasis is necessary to maintain normal life activities of cells, tissues and organs. Abnormal iron accumulation disrupts iron homeostasis, leading to the overproduction of ROS through the Fenton reaction, which in turn exacerbates tissue injury through inflammation, ferroptosis, tissue scar repair and other unfavorable reactions. In addition, ROS acts as signaling molecules to regulate iron homeostasis by affecting the activity of regulators of iron metabolism, which may result in a new form of cell death, ferroptosis. This may be one of the mechanisms of how abnormal iron metabolism and oxidative stress cause renal injury. 

Understanding the underlying mechanisms of iron metabolism and oxidative stress in kidney diseases is crucial to discovering possible therapeutic targets and devising effective methods to reduce the burden of kidney diseases. The existence of a vicious cycle of abnormal iron metabolism and oxidative stress perpetuate each other. Further investigation into the correlation between iron metabolism and oxidative stress in the setting of kidney disease is crucial, as it can offer valuable perspectives on potential therapeutic approaches. Future studies need to delve into the following concerns, including verifying (1) besides the Fenton reaction, whether there are any other ways for iron ions to regulate ROS production, and (2) in addition to ROS leading to extreme oxidative stress, whether there are any other mechanisms in ROS to alter cellular phenotypes. Because the mechanisms related to ferroptosis are poorly defined, it is necessary further explore novel molecular pathways and regulatory mechanisms related to ferroptosis. Although there is a possible association between iron deposition and kidney injury in hemochromatosis, more in-depth studies are needed to explore whether and how dysregulation of iron metabolism (iron deposition or iron deficiency) directly leads to kidney injury and impaired renal function. In addition, thus far, almost all mechanistic studies are based on experimental animals. The individual differences between humans and experimental animals should be verified in a clinical setting. In sum, an in-depth understanding of the iron metabolic pathways and the regulatory mechanism of oxidative homeostasis is of great interest in determining their specific roles in various kidney diseases and discovering the corresponding safe therapeutic targets for treatment. 

## Figures and Tables

**Table 1:** The classification of ROS.

	Reactive Oxygen Species	Properties
Non- radical	Hydrogen peroxide (H_2_O_2_)	Low oxidative activity, participates in many physiological processes as a signal molecule
	Organic hydroperoxides (ROOH)	Lipid peroxides derived from polyunsaturated acids (PUFAs) in ferroptosis
	Singlet oxygen	High oxidative activity involved in many biological processes
	Electronically excited carbonyl (R–C=O)	High oxidative activity
	Peroxynitrite (ONOO^−^)	Formed by the reaction of superoxide with nitric oxide
	Ozone (O_3_)	In atmosphere, but toxic to humans
Free radical	Superoxide (O_2_^−^)	Relatively low oxidative activity, participates in the synthesis of H_2_O_2_
	Hydroxyl radicals (HO∙)	High oxidative activity and unstable, react with various cellular proteins, DNA, lipids
	Peroxyl radical (ROO∙)	High oxidative activity, involved in the spread of lipid peroxidation
	Nitric oxide (NO∙)	Relatively low oxidative activity, participates in the synthesis of H_2_O_2_

**Table 2:** The enzymatic and non-enzymatic renal antioxidant systems.

Enzymatic System	Non-Enzymatic System
Superoxide dismutase (SOD)	Glutathione (GSH)
Catalase (CAT)	Antioxidant vitamins: vitamin A/C/E
Glutathione peroxidase (GPX)	Antioxidant minerals: copper, zinc, manganese
Thioredoxin (Trx)	Hormones: melatonin, flavenoids,
	coenzyme Q

## Data Availability

Not applicable.
